# Impacts of thermal fluctuations on heat tolerance and its metabolomic basis in *Arabidopsis thaliana*, *Drosophila melanogaster*, and *Orchesella cincta*

**DOI:** 10.1371/journal.pone.0237201

**Published:** 2020-10-29

**Authors:** Natasja Krog Noer, Majken Pagter, Simon Bahrndorff, Anders Malmendal, Torsten Nygaard Kristensen

**Affiliations:** 1 Department of Chemistry and Bioscience, Aalborg University, Aalborg, Denmark; 2 Department of Science and Environment, Roskilde University, Roskilde, Denmark; Florida Agricultural and Mechanical University, UNITED STATES

## Abstract

Temperature varies on a daily and seasonal scale and thermal fluctuations are predicted to become even more pronounced under future climate changes. Studies suggest that plastic responses are crucial for species’ ability to cope with thermal stress including variability in temperature, but most often laboratory studies on thermal adaptation in plant and ectotherm organisms are performed at constant temperatures and few species included. Recent studies using fluctuating thermal regimes find that thermal performance is affected by both temperature mean and fluctuations, and that plastic responses likely will differ between species according to life strategy and selective past. Here we investigate how acclimation to fluctuating or constant temperature regimes, but with the same mean temperature, impact on heat stress tolerance across a plant (*Arabidopsis thaliana*) and two arthropod species (*Orchesella cincta* and *Drosophila melanogaster*) inhabiting widely different thermal microhabitats and with varying capability for behavioral stress avoidance. Moreover, we investigate the underlying metabolic responses of acclimation using NMR metabolomics. We find increased heat tolerance for *D*. *melanogaster and A*. *thaliana* exposed to fluctuating acclimation temperatures, but not for *O*. *cincta*. The response was most pronounced for *A*. *thaliana*, which also showed a stronger metabolome response to thermal fluctuations than both arthropods. Generally, sugars were more abundant across *A*. *thaliana* and *D*. *melanogaster* when exposed to fluctuating compared to constant temperature, whereas amino acids were less abundant. This pattern was not evident for *O*. *cincta*, and generally we do not find much evidence for similar metabolomics responses to fluctuating temperature acclimation across species. Differences between the investigated species’ ecology and different ability to behaviorally thermoregulate may have shaped their physiological responses to thermal fluctuations.

## Introduction

The natural environment is constantly changing and abiotic factors fluctuate continuously on various spatial and temporal scales. These changes can pose stress on organisms [1, 2] and especially extreme temperatures are important physical factors that affect the abundance and distribution of species [3–6]. To survive and reproduce in fluctuating and periodically stressful environments individuals and populations need to adjust their physiology, morphology or behavior to mitigate adverse effects on fitness. This can occur via phenotypic changes within the lifetime of an individual, cross-generational epigenetic responses, or evolutionary adaptations across generations [4, 7]. Most laboratory studies on physiological and evolutionary adaptation to various thermal conditions on plant and animal ectotherm species have used constant temperatures [2, 8]. However, under natural conditions temperatures are rarely constant and even less so in the face of climate change causing increased variability and decreased predictability of temperatures [9, 10]. Recent studies on *Drosophila* [11–17] and various other invertebrate species [18–23] have shown that thermal variability can have significant impacts on thermal performance and that conclusions drawn on the basis of studies in constant temperature environments does not always hold under variable thermal conditions. The impact of thermal fluctuations on stress resistance has been attributed to the convex shape of the performance curve. Temperature fluctuations will create a disproportionate increase or decrease in performance because the curve accelerates slowly from the lower extreme, gets steeper towards optimum performance, and decelerates rapidly when approximating the upper extreme. The acclimation effect will thus depend on the mean temperature and amplitude of fluctuations used in the study, a relationship known as Jensen’s inequality [1, 24, 25]. However, the empirical support for the theory has been inconsistent and likely depends on the past selective pressures on species and traits and the nature of thermal regimes applied [12, 26].

A common expectation for studies examining the effect of thermal variability on heat and cold stress resistance is that temporal variability in habitat temperatures will select for high levels of plasticity in thermal tolerance traits compared to stable thermal environments [27–30]. This is expected if the environmental cues for temperature changes are reliable [31, 32; but see 33], the heterogeneity occurs at a timescale relevant for the lifespan of the organism, and genetic variation for plasticity is present [32–36]. However, findings on the relationship between the latitudinal origin of populations and the level of plasticity in thermal tolerance are generally contrasting for both arthropod and plant species. Some studies find no relationship between latitude and plasticity for animal ectotherm species [16, 17, 37–39] and plants [40]. Other studies have found such an association for cold [41, 42] and heat tolerance [43, 44] in animals and for cold [45], and heat tolerance [46] in plants. Only few studies have examined geographic clines in thermal tolerance for plant species, but studies find associations between latitude and plasticity of other fitness-related traits, which suggest that climatic variability has a strong effect on plasticity in plants [47–51]. Despite only few studies finding a strong association between plasticity and temperature variability, there is a trend for broader thermal range with latitude that might be linked to differences in plasticity levels (Reviewed by Addo-Bediako et al. [52]; Angilletta [53]; Sunday et al. [54]; see also [16, 55]; but see [37]). Yet, most studies find that this is caused by differences in inherent lower lethal limits, whereas upper thermal limits show little differentiation with latitude [38, 53, 56, 57; but see 58].

The discrepancies between findings on plasticity of thermal tolerance and thermal range (especially upper thermal limits) in different populations and species have been attributed to differences in the availability of microhabitats in terrestrial environments, and the capabilities for behavioral thermoregulation between e.g. plants and animal ectotherm species, and different life stages [54]. For instance, a stable microhabitat such as the soil environment will be buffered from large fluctuations in temperature and lower levels of plasticity may therefore be expected in soil living organisms such as collembolans [59, 60]. Despite this expectation, it has been demonstrated that differences in plasticity exists between local populations and different species [41, 43, 60, 61]. However, inducible heat tolerance caused by plasticity in the springtail *Orchesella cincta* is much slower compared to species such as *D*. *melanogaster*, which is likely due to the daily thermal fluctuations experienced by each species [59, 62].

In addition to microclimates, thermoregulatory behavior also reduces the temperature variation experienced by some invertebrates because they actively seek shade, bask in the sun, or move between sun and shade [63, 64]. The capacity for behavioral thermoregulation will depend on the mobility of the species as shown for different life-stages of invertebrates that differ in mobility levels and plastic responses in thermal limits [65–67]. For instance, for *D*. *melanogaster* the relatively immobile larval and pupal stages show much higher plastic change in survival to heat stress after heat hardening treatments compared to the mobile adult stage [68]. These findings suggest that mobility levels are important determinants of plasticity in nature and that plasticity becomes less important as adults gain the ability to avoid high temperatures. Likewise, plants have limited capability of thermoregulatory behavior on a short time scale (though some regulation occurs e.g. via changes in leaf and flower orientation and leaf rolling) and this can explain why plant studies generally find stronger associations between plasticity levels and latitude compared to studies on ectotherms [69, 70].

The sessile life strategy of plants might thus promote selection on alternative morphological, physiological, or biochemical mechanisms of stress avoidance and mitigation [49, 71–73]. Some core cellular stress responses that have been identified and functions across most taxa and environments, include molecular chaperone activity, changes in membrane lipid composition, altered energy metabolism, and build-up of cryoprotectants/osmoprotectants (Reviewed by Feder & Hofmann [74]; Hazel [75]; Kültz [76]). The importance of these mechanisms during thermal fluctuations is however poorly understood [1]. A valuable tool for understanding mechanisms underlying acclimation processes in plants and animals is metabolomics which provide an integrated measure of regulatory processes at the different molecular levels combined with external environmental influences [77, 78]. All of these processes are reflected in the metabolome, which is closely linked to the observed functional phenotype. Thus, the metabolome might constitute a reliable predictor of organismal phenotypes and provide novel insight into the underpinnings of complex traits such as responses to thermal fluctuations [79, 80].

Here, we investigate tolerance to heat stress in the arthropods *D*. *melanogaster and O*. *cincta*, and the plant *Arabidopsis thaliana*. We acclimated the three species at a constant and fluctuating temperature regime both with a mean of 20.4°C. We hypothesize that individuals exposed to thermal fluctuations will have higher heat tolerance compared to those exposed to constant thermal acclimation conditions prior to testing due to Jensen’s inequality. We further expect that the plastic response in heat tolerance to thermal fluctuations will differ between species because they inhabit thermally distinct environmental niches and have varying levels of mobility. Thus we anticipate a strong plastic response to thermal fluctuations in the immobile plant *A*. *thaliana*; a moderate response in the vinegar flies, *D*. *melanogaster*, where the adult life-stage is readily mobile but occupying temperature variable environments especially in immobile egg and pupae stages; and lowest response in collembolans, *O*. *cincta*, that occupy a buffered soil environment. We further investigate the metabolomic consequences of exposure to respectively constant and fluctuating temperatures using NMR metabolomics hypothesizing that both shared and distinct responses to thermal fluctuations are observed across the three species.

## Materials and methods

### Ethics

No endangered or protected species were included in the present study. The collembolans were collected in a public park with no specific permission required for collection and flies were collected at a private farm with permission from the land owner.

### Species and populations used for experiments

The experiment was performed in two independent experimental runs with sampling for NMR metabolomics in the second run. The chosen species represent distinct life strategies and microhabitats.

#### Drosophila melanogaster

Flies used in the study were from a population of *D*. *melanogaster* that was set up in 2010 using the offspring of 589 inseminated females caught at Karensminde fruit farm in Odder, Denmark (for further details see [81]). The population was maintained in the laboratory for ca. 220 generations at a population size >1000 individuals prior to performing the experiments reported here. The flies were held in plastic bottles containing 50 mL agar-sugar-yeast-oatmeal standard *Drosophila* medium [82] at a density of approximately 200 flies per bottles and maintained at 19°C in a 12:12 h light/dark regime. Experimental flies were produced by density-controlled egg laying in 10 bottles of 200 flies for 6 hours. Newly eclosed flies were transferred to bottles with fresh media within 12 hours of eclosion. One day prior to acclimation start the flies were sexed under light CO_2_ anesthesia (<5 min) and 180 groups of 10–12 males (only male flies were used for the experiments) were placed in 35 mL vials containing 7 mL standard *Drosophila* medium. Flies were 5–6 days of age at acclimation start for both experimental runs. During acclimation, fresh media was provided every other day.

#### Orchesella cincta

Collembolans used in the experiment originated from a population collected in Siena, Italy, in 2016 and thereafter maintained at 20°C, 70% RH and a photoperiod of 12:12 hour light: dark regime for ca. 15 generations (for details see [40]). During acclimation, 10–13 collembolans of unknown sex were held in 180 replicate petri dishes (55 mm) containing a water-saturated plaster-of-paris: charcoal (9:1) medium and an algae-covered twig was provided as food source. A few drops of water were added to each petri dish every day to prevent desiccation stress and fresh food was provided every other day. Collembolans used for the experiment were 8 weeks old at acclimation start in the first experimental run and 10 weeks old at the second run. Heat tolerance in *O*. *cincta* within this age-span has been shown not to differ [83].

#### Arabidopsis thaliana

*A*. *thaliana* seeds used in the study were from the Columbia-0 (Col-0) accession. Seeds were surface sterilized in 70% (v/v) ethanol for 10 min and then briefly mixed with 100% (v/v) ethanol before drying on a piece of sterile filter paper. Surface-sterilized seeds were stratified in sterile water at 4°C for three days prior to plating on 55 mm petri dishes containing 1x Murashige and Skoog (MS) basal growth medium (Duchefa), 0.5 gL^-1^ MES and 1.0% (w/v) agar at pH 5.7. Seeds were plated at a density of 24 seeds per plate on 180 replicate plates. Plates were sealed with Micropore tape and placed horizontally in a growth room at 20°C with an 16 h day length at 150 μmol m^-2^ s^-1^ for three days to allow seedlings to emerge, after which the acclimation treatments were initiated.

### Thermal acclimation regimes

**Fig 1:**
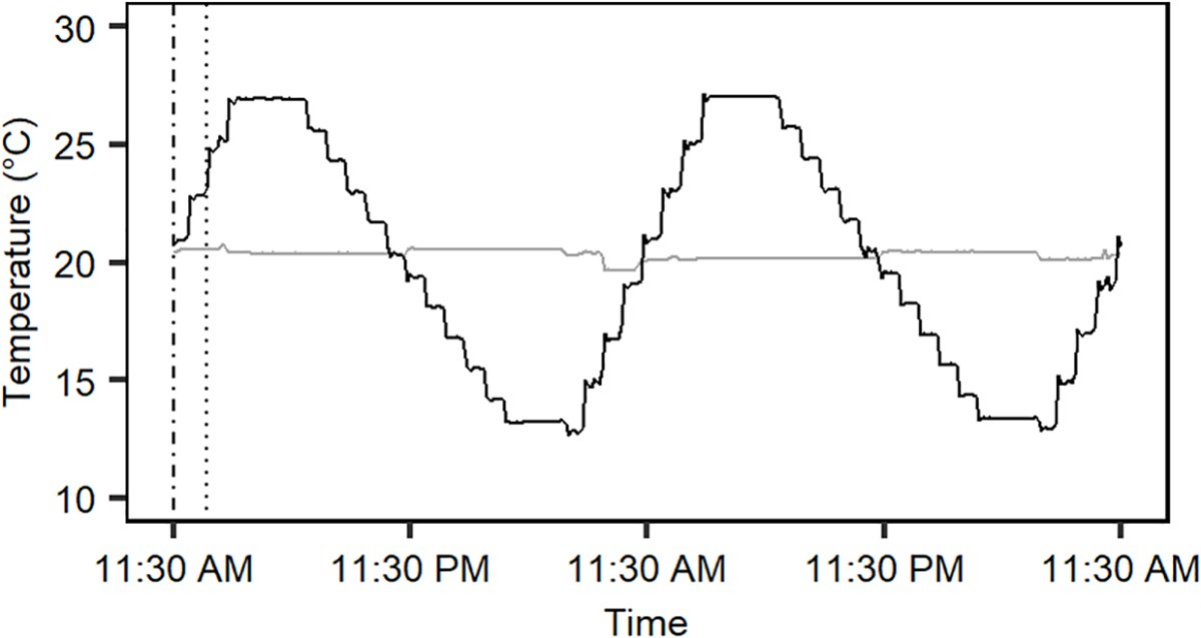
Thermal acclimation regimes. Section of the recorded temperatures in the constant (grey) and fluctuating (black) thermal regimes from the second experimental run. The constant thermal regime maintained a temperature of 20.4± 0.2°C and the fluctuating regime cycled around the mean temperature in intervals of 13.2–26.9°C. Adult flies, collembolans and plant seedlings were acclimated to each acclimation regime for 6–7 days. The experiment was performed twice and the dotted and dashed lines represent the temperature at the initiation of acclimation treatments in the first and second experimental run, respectively.

A constant and a fluctuating thermal regime with equal mean temperature and a photoperiod of 16:8 h light/dark and a light intensity of 150 μmol m^-2^ s^-1^ that resemble Danish summer conditions were generated in two programmable Plant Growth Chambers (Snijders Microclima MC1750E). These long-day conditions were used to meet the requirements for plant growth and animals were kept under the same conditions to ensure comparable results. The constant regime retained a temperature of 20.4± 0.2°C throughout the day and night. The fluctuating thermal regime varied predictably and diurnally around the mean temperature 20.4°C, reaching a minimum temperature of 13.2± 0.1°C, at rate of 0.04°C min^-1^, early in the morning, and a maximum temperature of 26.9± 0.1°C, at a rate of 0.06°C min^-1^, in the afternoon (see Fig 1).

Exposure to the acclimation treatments (constant or fluctuating) were started by relocating 90 replicate petri dishes (*A*. *thaliana* and *O*. *cincta*) and vials (*D*. *melanogaster*) for each species randomly to the two acclimation treatments. The temperatures in the constant and fluctuating regimes were 20.4 and 24.3°C, respectively, at acclimation start for all organisms in the first experimental run, and 20.4°C in both regimes in the second run (Fig 1). Differences in start temperature in the two regimes in run 1 (which was not intended) were considered negligible due to the long acclimation exposure periods. The replicate petri dishes and vials were reshuffled in a randomized manner inside the chambers once a day during the acclimation period to minimize slight internal differences in temperatures and light that individual replicates may have been exposed to in the chambers. Flies and collembolans were acclimated for 7 days, and seedlings for 6 days before assessment of their heat tolerance and freezing of individuals for NMR (from second experimental run).

### Heat tolerance

Heat tolerance was tested using a heat mortality assay exposing the organisms to 7–8 species specific temperatures ranging from non-lethal to lethal: *D*. *melanogaster* were exposed to 35, 36, 37, 38, 39, 40, 41°C; *O*. *cincta* to 35, 36, 37, 38, 39, 40, 41, 42°C; *A*. *thaliana* to 37, 39, 41, 43, 44, 45, 47, 49°C. The species-specific test temperatures were based on pilot tests (results not shown). Thermal incubators were used to generate the different test temperatures and the tests were conducted at midday when the temperature in both thermal regimes was ~20.4°C. This was done to minimize effects of different temperatures and daily rhythm on heat tolerance. For flies and collembolans, 10 randomly chosen replicates of 10 individuals from each acclimation treatment were exposed to each stress temperature for 1 hour. During the tests the flies were kept in plastic vials containing 7 mL standard *Drosophila* medium and collembolans were kept on Plaster-of-Paris medium. Following exposure to heat stress, *D*. *melanogaster* and *O*. *cincta* recovered for 1 hour at 20.4°C and subsequently the mortality was scored as the number of dead individuals out of the total number of individuals in each replicate. For *A*. *thaliana*, 10 randomly chosen replicate plates with ~24 seedlings from each acclimation treatment were likewise exposed to each of the chosen test temperatures for 1 hour and then returned to the growth chamber at a constant temperature of 20.4°C. The seedlings were kept in the petri dishes containing (MS) medium during the test and recovery period. Thermal damages in plants build up slowly when exposed to moderately high temperatures and to ensure that we had accounted for the total mortality caused by thermal stress the number of viable seedlings was quantified after 7 days of recovery. Seedlings that were still green and produced new leaves were scored as survivors.

### NMR

In the second experimental run, we repeated the experimental design and stress tests described above, but prior to stress tests randomly chosen samples of arthropods and plants from each acclimation treatment were snap-frozen in liquid nitrogen for later NMR metabolomic analysis. Metabolites were extracted from 6 replicates of 10 male flies and 6 replicates of 10 non-sexed collembolans from each acclimation treatment using the same protocol as described by Ørsted et al. [84] and from 5 replicates of 24 seedlings from each acclimation treatment for *A*. *thaliana*. In short, whole-body tissues from each sample were mechanically homogenized in 1 mL of Acetonitrile solution (50%, 50% ddH_2_O) using sterile glass beads and a homogenizer (FastPrep-24^TM^—MP Biomedicals) for 2 x 35 sec at 3800 rpm. The plant samples were further sonicated for 15 minutes at room temperature prior to the proceeding steps. All samples were cooled on ice, centrifuged at 14,000 rpm for 10 min at 4°C. The supernatant was transferred to new tubes, snap frozen, lyophilized, and stored at -80°C until NMR analysis.

NMR measurements were performed at 25°C on a Bruker Avance III HD 800 spectrometer (Bruker Biospin, Rheinstetten, Germany), operating at a ^1^H frequency of 799.87 MHz, and equipped with a 3 mm TCI cold probe. ^1^H NMR spectra were acquired using a standard NOESYPR1D experiment with a 100 ms delay. A total of 128 transients of 32 K data points spanning a spectral width of 20 ppm were collected. The spectra were processed using Topspin (Bruker Biospin, Rheinstetten, Germany). An exponential line-broadening of 0.3 Hz was applied to the free-induction decay prior to Fourier transformation. All spectra were referenced to the DSS signal at 0 ppm, manually phased and baseline corrected. The spectra were aligned using icoshift [85]. The region around the residual water signal (4.87–4. 70 ppm) was removed in order for the water signal not to interfere with the analysis. The high- and low-field ends of the spectrum, where no signals except the reference signal from DSS appear, were also removed (i.e., leaving data between 9.7 and 0.7 ppm).

### Data analysis

#### Thermal tolerance

We fitted a generalized linear model with a binomial link function on survival proportions for each species. This allowed us to look for batch effects between repeated experiments. A likelihood ratio test (LRT) was used to compare a model containing an interaction term between temperature and experimental run with a reduced model omitting this term. All LRTs were significant which indicated significant effects of experimental run and the data was treated as two independent experiments for the rest of the analysis.

For every heat exposure temperature, survival was calculated as the number of survivors over total number of individuals for each replicate. The Lethal median Temperature (LT_50_) for each acclimation treatment was found by logistic regression on survival proportions for each stress temperature using the drc-package in R [86]. Significant differences in LT_50_ for each species were found by comparing confidence intervals of LT_50_ estimates and by chi-square LRT on a model incorporating differences in LT_50_ between the two thermal regimes and a model assuming common LT_50_ for both.

#### NMR data

Multivariate analyses were performed on spectral data that was normalized by probabilistic quotient area normalization [87] to suppress separation caused by variation in sample volumes, and pareto-scaled to reduce variance caused by metabolite differences.

Principal component analysis (PCA) was used to differentiate metabolite content between species and thermal acclimation treatment. PCA analyses were run on the normalized and scaled spectral data in R using the built-in R function prcomp(). The analysis was run on the complete metabolite spectra dataset and on subsets containing individual species data. Significant effects of species and acclimation regime were tested on PCA scores using MANOVA in R [88].

Metabolome differences caused by thermal acclimation regime were further assessed using Orthogonal Projection to Latent Structures Discriminant Analysis (OPLS-DA) on the data combined for all species and for individual species. Validation scores for the OPLS-DA models were calculated by 7-fold cross-validation. The analysis is regression-based and seeks to correlate sample variation with a response vector that contains sample information (acclimation treatment) while finding uncorrelated variation (orthogonal components) that are systematic in the data. This analysis is useful when the effects of interest are masked by variables that have larger influences on the sample variation, e.g. species metabolome variation [89, 90]. OPLS-DA was carried out using the SIMCA16 software (Umetrics, Malmö, Sweden).

In order to identify the significant changes in metabolite concentrations when going from constant to fluctuating acclimation regimes the OPLS-DA loadings (amplitude and correlation) were plotted for all models. The correlations were calculated after removing the variation explained by the orthogonal components. The threshold for significant change in metabolites between acclimation treatments were *P* < 0.05 after correction for multiple testing for 50 metabolites. Relative changes in metabolite concentrations in organisms acclimated to the two thermal regimes were calculated for every significant metabolite as the difference in concentration in individuals from the constant and the fluctuating acclimation treatments divided by the median concentration in individuals from the constant acclimation regime.

## Results

### Thermal fluctuations increase heat tolerance

**Fig 2:**
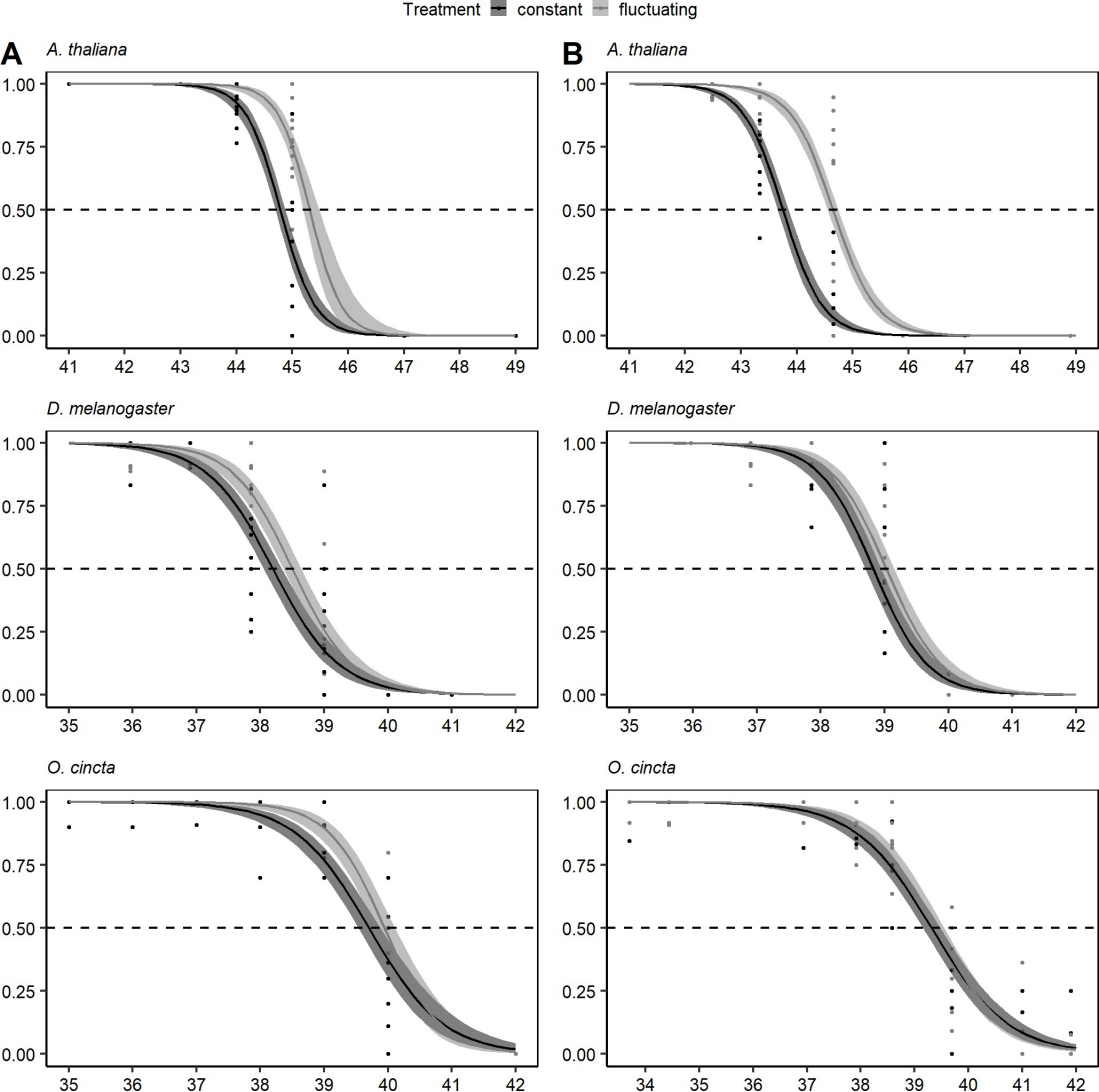
Survival proportions after heat exposure. Regression on survival proportions after exposure to heat stress at species-specific test temperatures for 1 hour for individuals acclimated to constant (black) and fluctuating (grey) thermal regimes. Panel A represents the first experimental run and panel B the second run. Individual points are survival proportion for each replicate and 95% confidence bands around the regression.

Acclimation to the fluctuating compared to the constant thermal regime with equal mean temperature consequently increased LT_50_ for *A*. *thaliana* and *D*. *melanogaster* in both experimental runs (Table 1, Fig 2). The heat tolerance of *A*. *thaliana* from the fluctuating thermal regime was 0.5°C higher in experimental run 1 and 0.9°C in run 2 (Table 1, Fig 2). For *D*. *melanogaster* these numbers were 0.3 and 0.2°C in run 1 and 2, respectively. A significantly higher LT_50_ was also observed in *O*. *cincta* from the fluctuating thermal regime where individuals had a 0.3°C higher LT_50_ in run 1 (Table 1, Fig 2). Generally, we observed higher heat tolerance for *A*. *thaliana* than for both invertebrates, whereas only slightly higher heat tolerance was observed in *O*. *cincta* compared to *D*. *melanogaster* (Table 1).

**Table 1:** Lethal median temperatures. LT_50_ values (mean ± SE) for species acclimated to constant and fluctuating thermal regimes. Significant differences in LT_50_ for the two acclimation treatments were found by chi-square test for each species.

Experimental run 1	LT_50_ (°C)		Chi-square test	
**Species**	**Constant**	**Fluctuating**	***p*-value**	***χ*^2^_(d.f)_**
***Drosophila melanogaster***	38.27 (±0.07)	38.56 (±0.07)	0.002	9.42_(1)_
***Orchesella cincta***	39.69 (±0.09)	39.94 (±0.08)	0.034	4.49_(1)_
***Arabidopsis thaliana***	44.79 (±0.05)	45.33 (±0.07)	< .001	53.71_(1)_
**Experimental run 2**	**LT_50_ (°C)**		**Chi-square test**	
**Species**	**Constant**	**Fluctuating**	***p*-value**	***χ*^2^_(d.f)_**
***Drosophila melanogaster***	38.82 (±0.06)	39.01 (±0.06)	0.028	4.81_(1)_
***Orchesella cincta***	39.30 (±0.08)	39.38 (±0.08)	0.460	0.55_(1)_
***Arabidopsis thaliana***	43.75 (±0.05)	44.64 (±0.05)	< .001	150.16_(1)_

### Effect of thermal fluctuations on the metabolome

**Fig 3:**
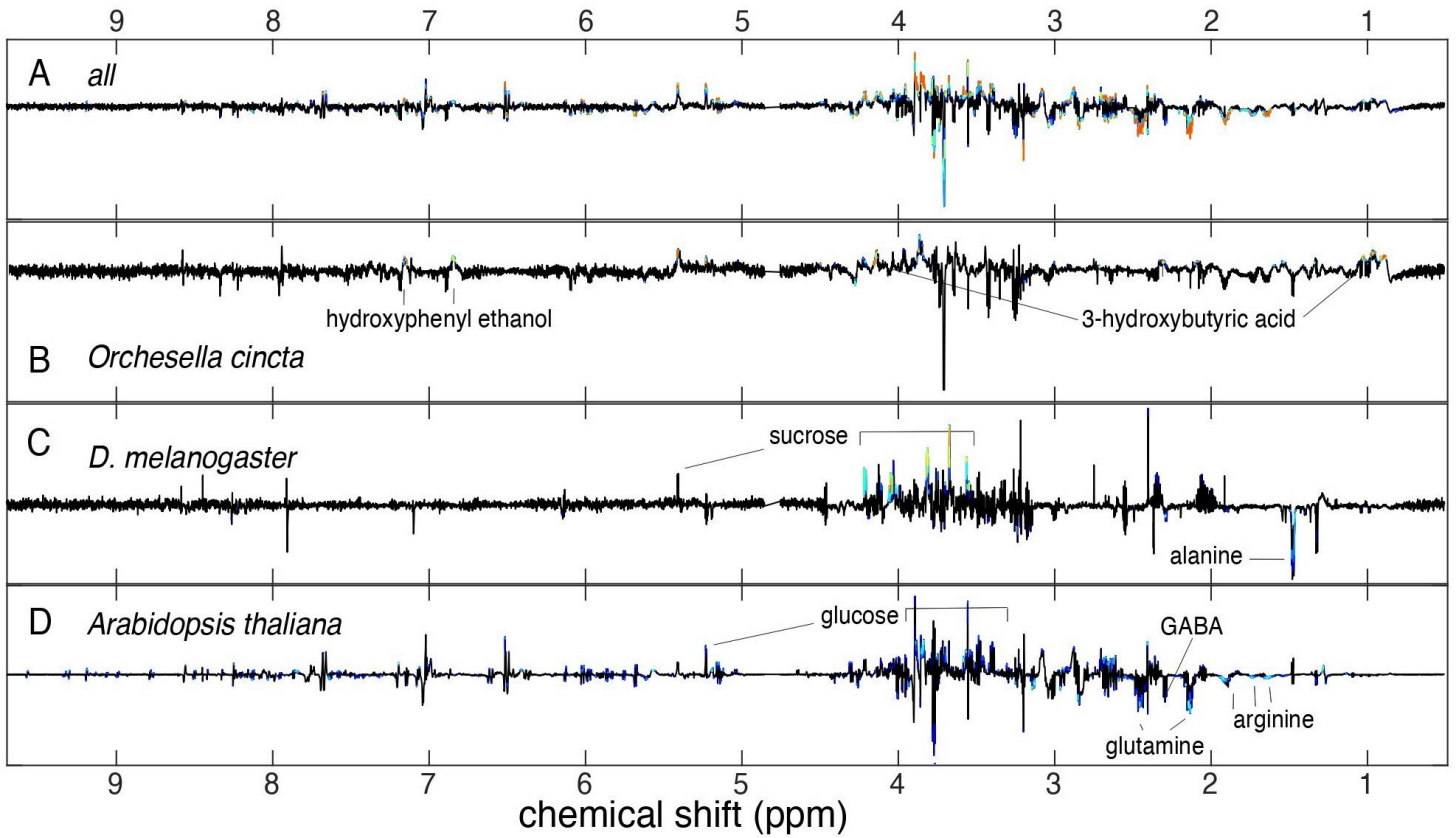
Metabolite loading spectra. Metabolite loading spectra showing significant (colored peaks, *p* < 0.05) up- or downregulated metabolites in individuals acclimated to fluctuating temperatures compared to constant temperature for A) layered spectrum for all organisms, B) *O*. *cincta*, C) *D*. *melanogaster*, and D) *A*. *thaliana*.

**Fig 4:**
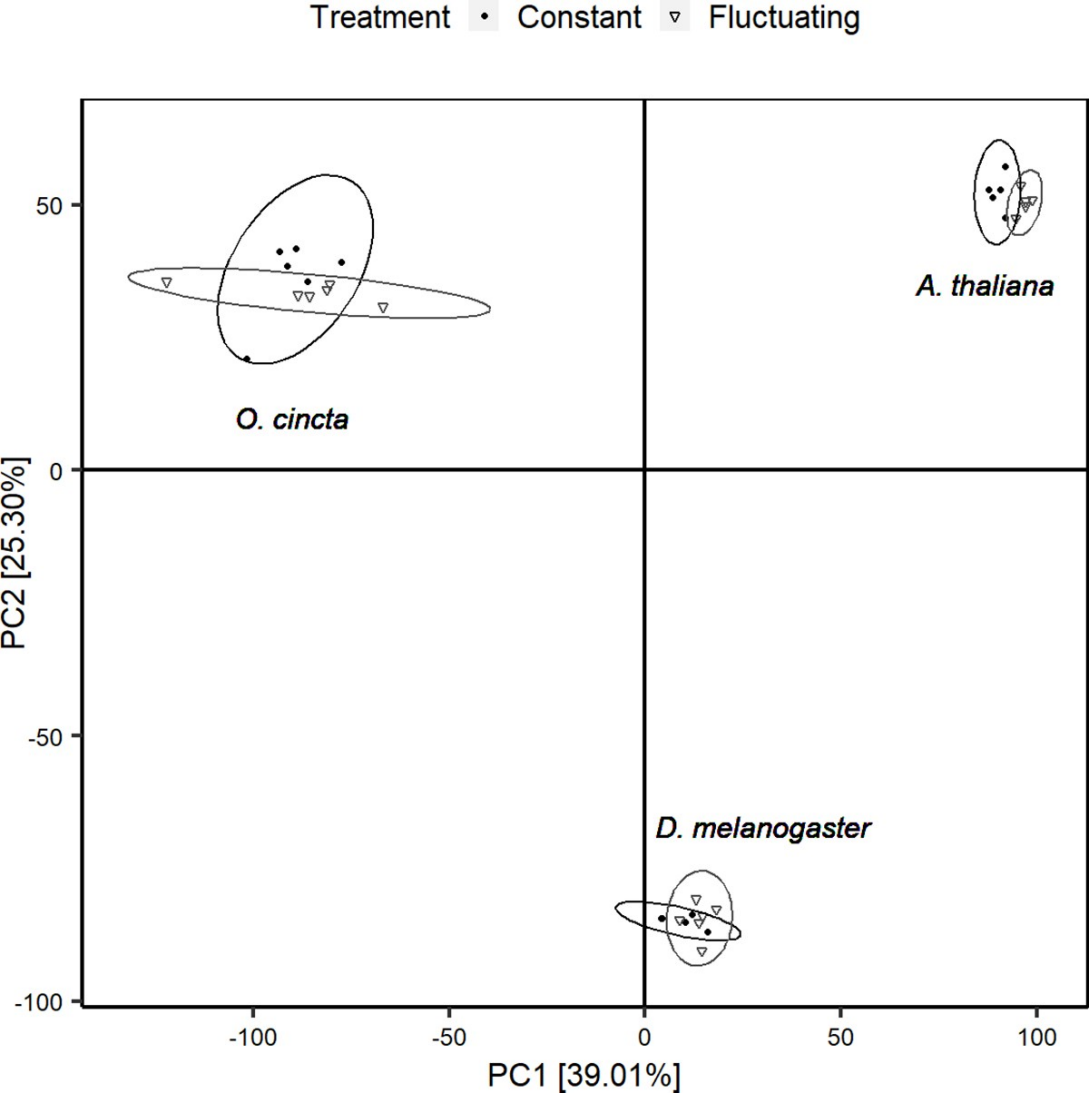
PCA scores plot. PCA on metabolite spectra from whole-body extract of *D*. *melanogaster*, *O*. *cincta*, and *A*. *thaliana* acclimated to constant (filled circles) and fluctuating (open triangles) thermal regimes. PC1 and PC2 account for 39.01% and 25.39% of the variance between samples, respectively.

**Fig 5:**
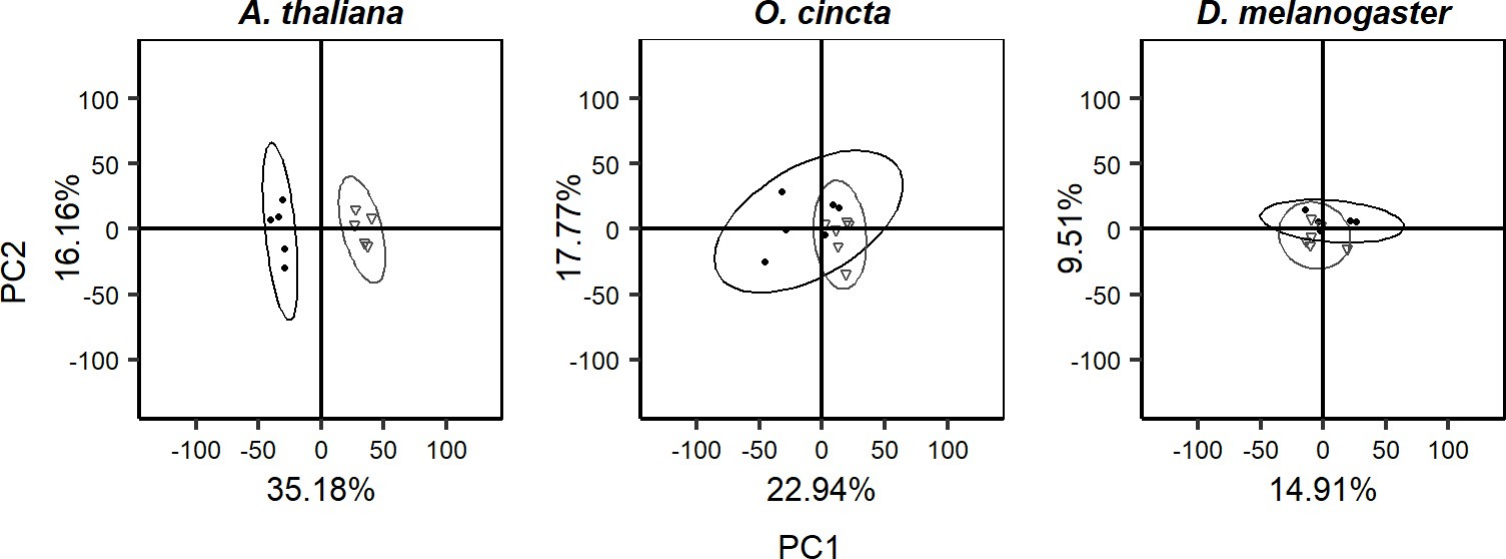
PCA scores for analysis on individual species. PCA scores plot for *A*. *thaliana*, *O*. *cincta*, *and D*. *melanogaster* acclimated to constant (filled circles) or fluctuating (open triangles) thermal regimes. Ellipses represent 95% CI.

Principal component analysis (PCA) was performed on the combined and separate metabolite spectra of *D*. *melanogaster*, *O*. *cincta*, and *A*. *thaliana* (Fig 3) to characterize the metabolite response underlying acclimation to constant and fluctuating thermal regimes for all species. The total variation between samples was explained by 33 principal components, however PC1 and PC2 accounted for most of this variation (64.3%, Fig 4 and S1 Fig). An inspection of the PCA scores plotted for PC1 and PC2 shows a distinct separation of metabolites associated with species and this was substantiated by a test for differential metabolite response on PCA scores (MANOVA; *p* < .001). PC1 further separates clusters associated with thermal regime (Figs 4 and 5), but this effect was not significant (MANOVA; *p* = 0.113).

Because of large variation caused by species-specific metabolite differences, an OPLS-DA was performed to focus the analysis towards differences in acclimation regime while diminishing variation caused by species. The OPLS-DA model was composed of one predictive component and three orthogonal components (S1 Table). Thermal regime accounted for merely 3% of the total metabolite variation in the samples, but the predictive ability of the model to correctly group a sample into constant or fluctuating acclimation treatment based on the metabolite content in the sample was significant (predictability Q^2^ = 0.6, S1 Table).

In addition, OPLS-DA models were performed on individual species (S1 Table). All models were of good quality, i.e. Q^2^ scores > 0.5. For *A*. *thaliana* 60% of the total metabolite variation was explained by the acclimation treatment, while the corresponding number was 18% for both *D*. *melanogaster* and *O*. *cincta* (S1 Table).

**Fig 6:**
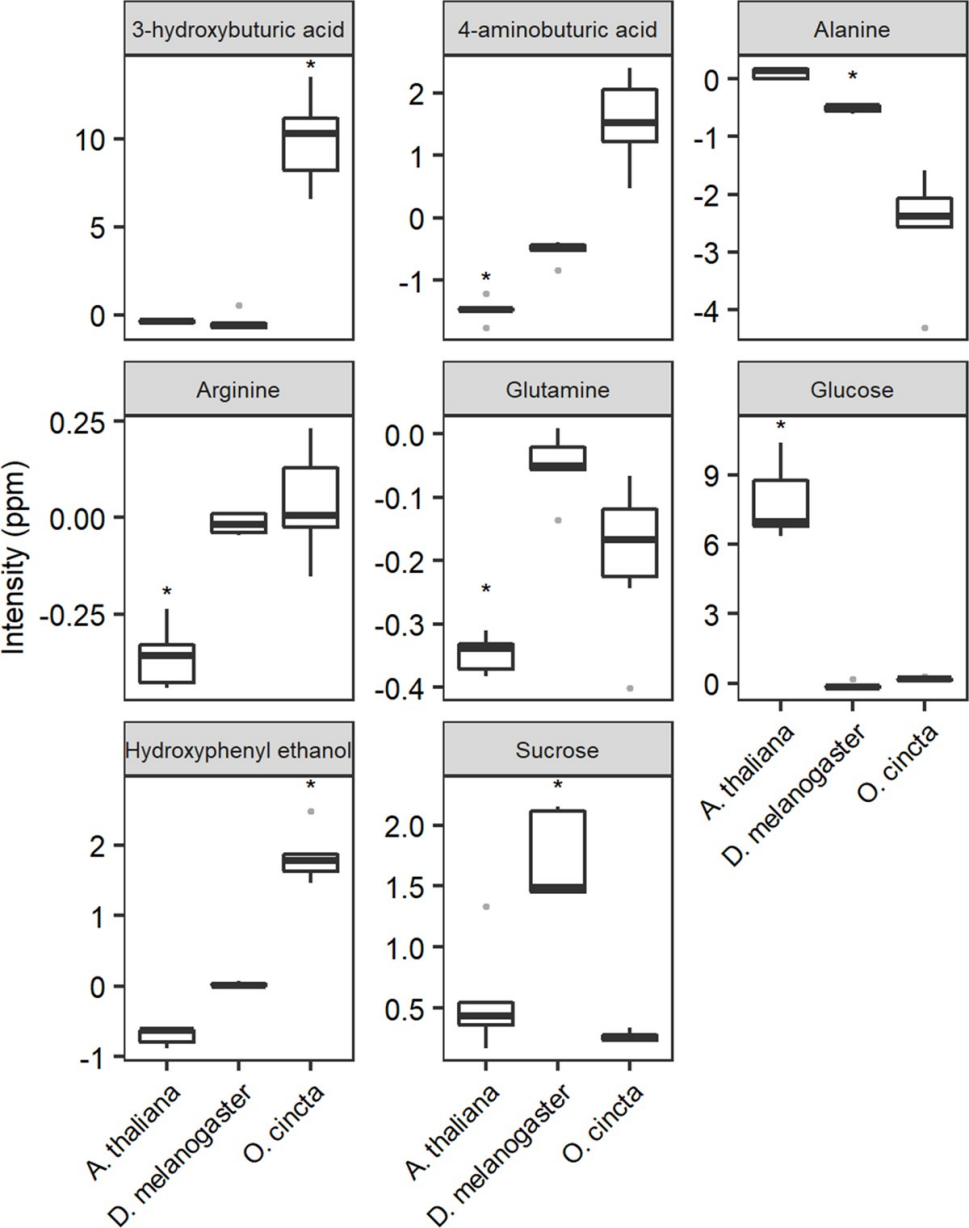
Relative change in concentrations of nine metabolites. Whisker box plots of change in relative concentrations of nine metabolites in *A*. *thaliana*, *D*. *melanogaster*, and *O*. *cincta* acclimated to fluctuating and constant temperatures, respectively. Significant changes in concentrations are indicated by * (*p* < 0.05). The y-axis shows the concentration of each metabolite in the organisms acclimated to fluctuating temperatures (measured as spectral intensity) relative to the mean concentration of the metabolites in organisms acclimated to constant temperatures. The centerline in each box represents the median, and the upper and lower boundaries are the 25^th^ and 75^th^ quantiles. The whiskers mark the extremes, and dots represent the outliers.

The OPLS-DA loadings from each individual OPLS-DA model were used to identify metabolites that differed significantly between individuals acclimated to fluctuating and constant thermal regimes (Figs 3 and 6). The analysis showed that the set of metabolites that was elevated or suppressed differed for each species. Metabolite changes that were significantly associated with the predictive component for *A*. *thaliana* included elevated levels of glucose and suppressed levels of glutamine (gln), arginine (arg), and gamma-aminobutyric acid (GABA) (Figs 3D and 6). Sucrose levels were elevated in *D*. *melanogaster* acclimated to thermal fluctuations and alanine (Ala) levels were lowered (Figs 3C and 6). Lastly, *O*. *cincta* that was exposed to thermal fluctuations had elevated levels of hydroxyphenyl ethanol and 3-hydroxybutyric acid (Figs 3B and 6).

## Discussion

In this study we investigated the effect of thermal fluctuations on heat tolerance and the metabolome in three taxonomically distant species. We found that individuals of *A*. *thaliana* and *D*. *melanogaster* acclimated at fluctuating temperatures were more heat tolerant than individuals acclimated to constant temperatures. This pattern was only observed for *O*. *cincta* in experimental run 1 (Table 1, Fig 2). The increased heat tolerance of individuals from the fluctuating temperature regime is in line with previous studies on *Drosophila* species exposed to predictable thermal fluctuations [11, 13, 23], but has to our knowledge not previously been shown for plants or collembolan species.

Of the three species that we investigated we observed the most pronounced increase in heat tolerance in response to thermal fluctuations for *A*. *thaliana* (Table 1, Fig 2). This finding suggests that *A*. *thaliana* has a larger capacity to adjust its phenotype to environmental fluctuations than *D*. *melanogaster* and *O*. *cincta*. Obviously we cannot make strong conclusions as to whether this result reflects general differences in responses to thermal fluctuations across species. However the observation agrees with studies on other plant species that have found strong correlations between latitudinal temperature variability and plasticity of various fitness-related traits, including photosynthetic rate, water-use efficiency, seed-output, leaf angles and number of flowers [46, 49–51, 91]. Such association between latitudinal temperature variability and fitness-related traits and heat tolerance has generally been much weaker for insects [16, 17, 37, 39, 92]. Although we cannot make any conclusions on why plants in general respond stronger to thermal fluctuation based on our study, it could be speculated that plants have developed stronger plastic responses as a consequence of their sessile lifestyle compared to invertebrates. We only found small differences in the acclimation response to constant and fluctuating temperatures for flies and an even weaker signal were observed for collembolans. Likewise, no significant effect of acclimation treatment on the metabolomes was found in these two species (Table 1 and Fig 4). This suggests a lower ability of these two species to induce a thermal plastic response which may relate to the ability of the species to behaviorally evade stressors in nature by e.g. seeking deeper into the soil column or escaping rapidly by flight or other types of maneuvering to more favorable thermal conditions [43, 61, 93]. This is supported by findings showing that sessile life stages of *Drosophila* are more plastic than adult stages [65, 66, 68]. However, studies generally find that collembolans show hardening responses to both cold and heat exposure, but this response is slow and maybe not working at the timescale of daily temperature fluctuations [41, 43, 59, 60].

The magnitude of the acclimation response observed in LT_50_ values was manifested in the metabolome showing bigger differentiation for *A*. *thaliana* exposed to fluctuating and constant temperatures compared to the arthropod species investigated (Figs 4 and 5, and S1 Table). This is well in accordance with the idea that this plant species, due to its limited mobility, exert a greater metabolite response to environmental fluctuations than invertebrates. Further, we found that the set of metabolites that was elevated or suppressed in response to thermal fluctuations for each species differed, but some of the affected metabolites shared some biochemical properties. For instance, sugar levels were elevated in *A*. *thaliana* and *D*. *melanogaster* exposed to thermal fluctuations, whereas amino acids were suppressed (Fig 6). These patterns were not found for collembolans, which showed a markedly different metabolite response than flies and plants.

Accumulation of soluble sugars, which act as compatible compounds that help stabilizing proteins and membranes and regulating osmotic pressure, is a common low temperature response in invertebrates [61, 93–96] and a low and high temperature response in plants [73, 97–100]. In our experiment, heat tolerance was tested at midday when temperatures in the fluctuating thermal regime had returned to initial mean temperature subsequent to a cool thermal peak reaching 13°C during the night. It has previously been found that *D*. *melanogaster* exposed to gradual cooling shows increasing levels of sugars and decreasing levels of amino acid when temperatures approximate 10°C and that the levels remain elevated for up to 4 hours after returning to pre-cooling temperatures [95]. Thus, it is likely that the accumulation of sugars we observed was an effect of cold ramping during the cold part of the thermal fluctuations which was maintained during the return to mean temperature. Accumulation of sugars during exposure to cold temperatures has been linked to the direct effect of low temperatures on enzyme activity involved in carbohydrate metabolism in invertebrates [101] and may also be the reason for the observed accumulation of sugars in this study.

Seemingly, sugars also accumulate in some plants exposed to temperature variation [102, 103]. A recent study found that natural changes in light and varying temperature have profound impact on daily rates of primary metabolism of *A*. *thaliana* compared to stable climate conditions with a constant temperature and sinusoidal simulations of light intensity [103]. Thus, most metabolites increased in the daytime and declined during the night in both the constant and variable thermal regimes, reflecting the build-up of reserves in the light and their consumption in the dark. However, the level of sugars and starch were higher at dawn in the naturally variable regime and this pattern was associated with slow carbon utilization at night due to cold temperatures. Thus, findings of changed levels of sugars for *A*. *thaliana* and *D*. *melanogaster* when exposed to fluctuating temperatures might not be a direct effect of accumulation of osmoprotectants, but rather indicate an indirect effect of altered metabolism such as slower utilization of energy-yielding molecules at night and faster synthesis at day.

Similar to findings in our study also Annunziata et al. [103] observed that acclimation of *A*. *thaliana* to a variable temperature regime resulted in lower levels of amino acids than in a stable temperature regime. This may partly be a result of decreased metabolic connectivity, which affected amino acids in particular or rapid incorporation of amino acids into proteins during high daily temperatures and slow protein degradation at night due to low temperature. These results can also explain the decreased levels of GABA for *A*. *thaliana* that was found in the fluctuating regime in our study. GABA is a non-protein amino acid that is found in both animals and plants and is proposed to be involved in intercellular stress signaling [104, 105]. However, the regulation and function of GABA is still uncertain and it is proposed that increases of GABA with thermal stress is simply a product of protein degradation [106, 107]. Thus, in accordance with the lower levels of free amino acids found in this study, the decreased levels of GABA might also reflect slow degradation of proteins at night when temperatures are low.

Findings of changed levels of sugars and amino acids for *A*. *thaliana* and *D*. *melanogaster* when exposed to fluctuating temperatures are interesting as they share some common responses to high and low temperature stress. However, in the current study, it is not possible to deduce whether these metabolites have a direct impact on heat tolerance or merely reflect indirect effects of altered metabolism. Future studies should focus on determining the separate and combined effects of the cold and warm temperatures that individuals are exposed to during thermal fluctuations on the metabolome and more in depth analyses of molecular mechanisms, pathways and connectivity between constituents.

In *O*. *cincta* an increase in 3-hydroxybutyric acid and hydroxyphenyl ethanol was found in response to fluctuating temperatures (Figs 3A and 6). The first is a ketone that is produced from fatty acid metabolism when cellular carbohydrate levels are low [108]. This could be an indication of increased energy demand, potentially because of increased metabolic rate as more time is spent at higher temperatures due to Jensen’s inequality, or alternatively due increased production of cellular protective molecules for stress mitigation. Hydroxyphenyl ethanol is a phenolic compound, which is synthesized by some organisms, including fungi, bacteria, and algae species which constitute the food items for collembolans [109]. Thus, the elevated levels of this metabolite presumably come from increased food consumption, maybe as a response to increased energy demand in the fluctuating compared to the constant thermal acclimation regime. Phenolic compounds that are ingested via foods possibly act as antioxidants [96] which protect cells from oxidative stress in humans [110]. High and low temperatures lead to cellular changes that induce oxidative stress [111] and thus, the increased levels of hydroxyphenyl ethanol found in collembolans (Fig 6) might help alleviating cells from oxidative stress. However, these are merely speculations and need further testing.

## Conclusion

Collectively, results of the present study reveal that *A*. *thaliana* and *D*. *melanogaster* show increased heat tolerance in response to acclimation to fluctuating temperatures in accordance with Jensen’s inequality. The response was generally low and would in terms of climate change probably have little impact on survival in nature. However, we only measured one aspect of heat tolerance and this conclusion might have been different if examining other measures of heat tolerance, different life stages of the organisms or other acclimation regimes. Despite this, we found a larger response of the plant species, and we speculate that this can be linked to its sessile life-style. However, this study did not include enough species belonging to different thermal habitats or with different levels of mobility to make any conclusions on this matter. The underlying metabolome response to acclimation was stronger in *A*. *thaliana* than in the invertebrates. The physiological mechanisms of adaptation were comprised of metabolites involved in primary metabolism in both *A*. *thaliana* and *D*. *melanogaster*, but showed a markedly different metabolite response in *O*. *cincta* involving fatty acid metabolism and phenolic compounds. We cannot conclude whether these differences in metabolomes have direct impact on heat tolerance or if different mechanisms were induced during acclimation and heat stress. Further studies might focus on finding differential responses before, during and after heat stress to determine if exposure to fluctuating, but non-stressful temperatures also changes the response to highly stressful conditions compared to acclimation at constant temperatures. This aspect is important to investigate to gain knowledge on whether exposure to fluctuating temperatures is adaptive or if the changes in metabolites are only indirect effects of rates of metabolism. stylefix.

## Supporting information

S1 Fig PCA scree plot. PCA scree plot from PCA analysis on metabolite spectra from whole-body extract of *D*. *melanogaster*, *O*. *cincta*, and *A*. *thaliana* acclimated to constant and fluctuating thermal regimes. PC1 and PC2 captures most of the inertia in the data.
(DOCX)

S1 Table OPLS model statistics for parameter prediction from metabolite data. The capability of the different metabolomes to predict the temperature regimes was tested using OPLS models.
(DOCX)
