# Supplementary figures and images for: Impacts of thermal fluctuations on heat tolerance and its metabolomic basis in Arabidopsis thaliana, Drosophila melanogaster, and Orchesella cincta
Source: PLoS One. 2020 Oct 29;15(10):e0237201. doi: 10.1371/journal.pone.0237201 (PMC7595314; doi:10.1371/journal.pone.0237201)

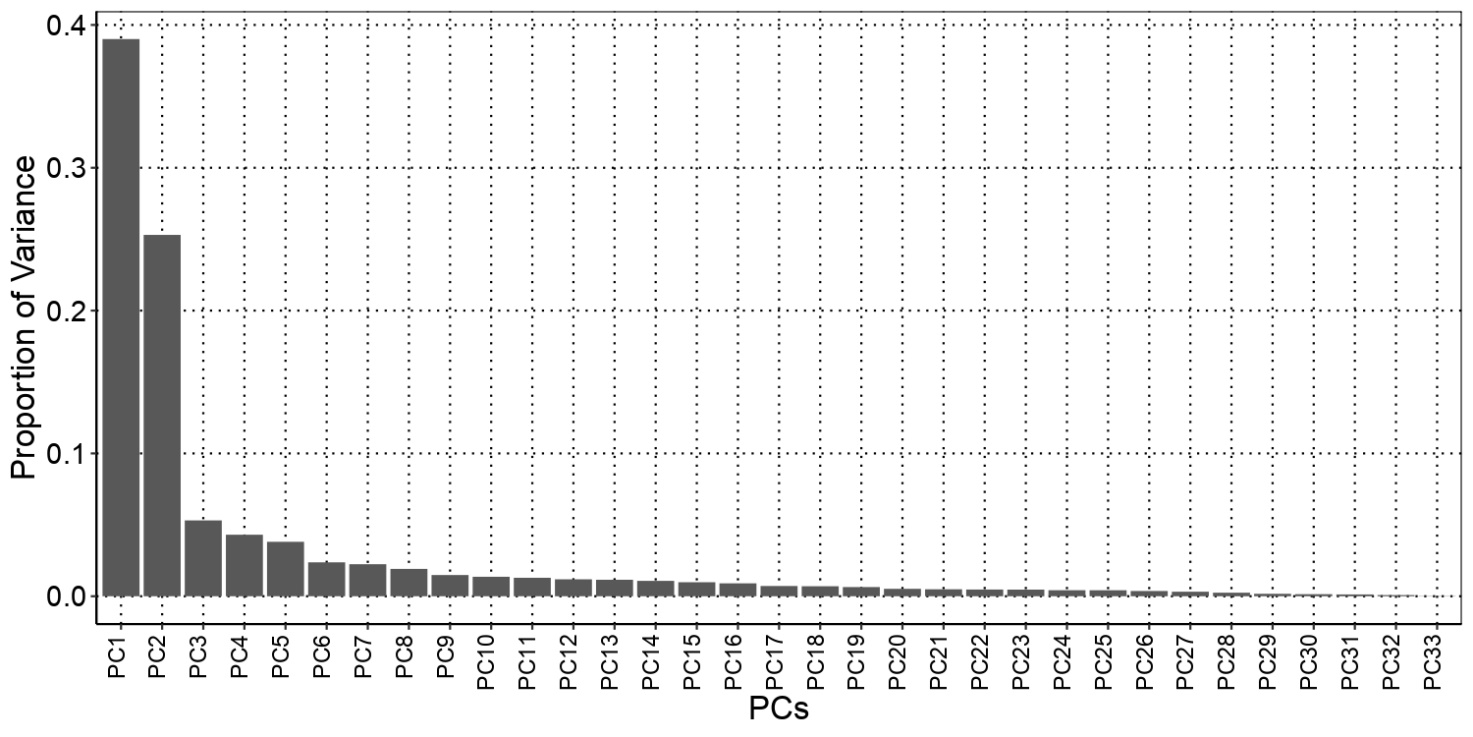

Supplement: S1 Fig — PCA scree plot from PCA analysis on metabolite spectra from whole-body extract of D. melanogaster, O. cincta, and A. thaliana acclimated to constant and fluctuating thermal regimes. PC1 and PC2 captures most of the inertia in the data. (DOCX) [file pone.0237201.s001.docx]
