# Supplementary material for: Impacts of thermal fluctuations on heat tolerance and its metabolomic basis in Arabidopsis thaliana, Drosophila melanogaster, and Orchesella cincta
Source: PLoS One. 2020 Oct 29;15(10):e0237201. doi: 10.1371/journal.pone.0237201 (PMC7595314; doi:10.1371/journal.pone.0237201)
Supplement: S1 Table — The capability of the different metabolomes to predict the temperature regimes was tested using OPLS models. (DOCX) [file pone.0237201.s002.docx]

| **Species** | **A^†^** | **N^‡^** | **R^2§^** | **R_p_^2*^** | **Q^2#^** |
| --- | --- | --- | --- | --- | --- |
| ***All*** | 1+3 | 33 | 0.91 | 0.03 | 0.60 |
| ***A. thaliana*** | 1+0 | 10 | 0.60 | 0.60 | 0.96 |
| ***D. melanogaster*** | 1+1 | 11 | 0.55 | 0.18 | 0.72 |
| ***O. cincta*** | 1+2 | 12 | 0.48 | 0.18 | 0.69 |

^†^A describes the number of model components where the first number accounts for the predictive component(s) correlating with the predicted variable, and the second the orthogonal component(s).

^‡^N describes the number of observations included in the model.

^§^R^2^ describes how much of the total metabolite variation that is explained by the model.

^*^R_p_^2^ describes how much of the total metabolite variation that is explained by the predictive component and thus depends on the temperature regime.

^#^Q^2^ describes the predictability of the total model and is related to the statistical validity of the model. Q^2^ ≥ 0.5 is considered significant. Q^2^ was calculated using 7-fold cross-validation.
